# Supplementary figures and images for: Crossmodal congruency effect scores decrease with repeat test exposure
Source: PeerJ. 2019 May 22;7:e6976. doi: 10.7717/peerj.6976 (PMC6535039; doi:10.7717/peerj.6976)

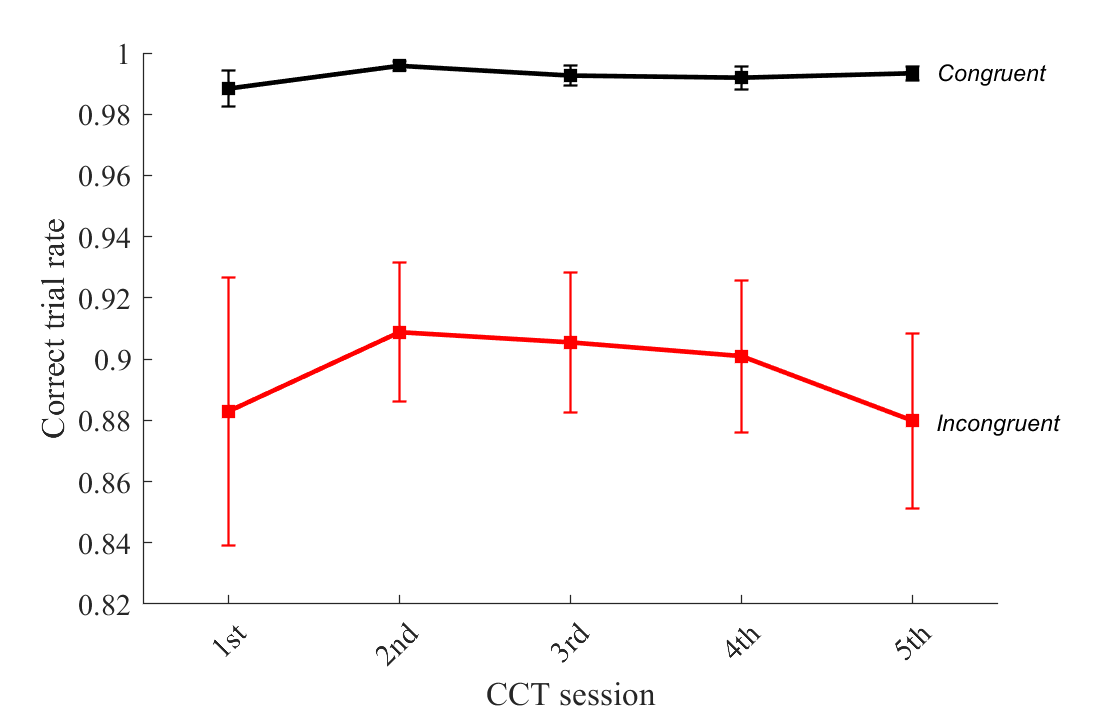

Supplement: Figure S1 [file peerj-07-6976-s001.png]

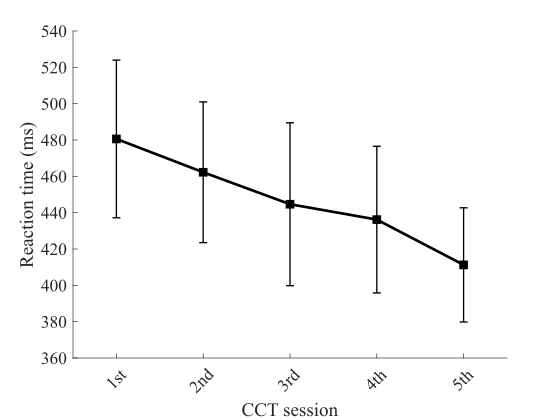

Supplement: Figure S2 [file peerj-07-6976-s002.png]
