# Supplementary material for: Crossmodal congruency effect scores decrease with repeat test exposure
Source: PeerJ. 2019 May 22;7:e6976. doi: 10.7717/peerj.6976 (PMC6535039; doi:10.7717/peerj.6976)
Supplement: Appendix S1 [file peerj-07-6976-s003.docx]

Appendix. Power analysis details

A power analysis was run to determine the number of sessions and subjects to include in Experiment 1. CCE scores from a pilot study of 8 subjects showed a mean of 125.6ms and a standard deviation of 47.5ms (σ).

CCE scores for two other pilot subjects were collected for 10 sessions each. The average of the 10 session CCE score data were fit with the least-squares function

$CCE_{i}=125.6*i^{-0.169}$ Eq. S1

where *i* is the session number and *CCE_i_* is the CCE score expected on the *i*^th^ session. The expected change in CCE score from the first session to the *i*^th^ session (*M_i_*) was calculated as

$M_{i}=CCE_{0}-CCE_{i}$ Eq. S2

The number of subjects required to detect an *M_i_* change in CCE score was computed as

$n=\left(\frac{z^{*}\sigma}{M_{i}}\right)^{2}$ Eq. S3

Where *z^*^* is the critical number for the confidence interval set to 1.96. Equation S3 was used to calculate the required number of subjects (*n)* necessary to detect the expected change in CCE score (*M_i_*) across up to 10 sessions with the indicated statistical power. The 5 session experiment requiring 10 subjects was selected for Experiment 1 (note we included 12 subjects to account for the small pilot study). The results are summarized in Table A1.

Table A1. Power analysis to determine subject number.

| i  Session # | n  Required number of subjects | M  Change in CCE from session 1 predicted by Eq S1 | σ  Standard deviation from pilot study | z*  Critical number for confidence interval |
| --- | --- | --- | --- | --- |
| 1 | -- | 0 | 47.5 | 1.96 |
| 2 | 45 | 13.88395041 | 47.5 | 1.96 |
| 3 | 20 | 21.28274576 | 47.5 | 1.96 |
| 4 | 13 | 26.23315497 | 47.5 | 1.96 |
| **5** | **10** | **29.91062705** | **47.5** | **1.96** |
| 6 | 9 | 32.81408004 | 47.5 | 1.96 |
| 7 | 7 | 35.2000749 | 47.5 | 1.96 |
| 8 | 7 | 37.21726603 | 47.5 | 1.96 |
| 9 | 6 | 38.95915978 | 47.5 | 1.96 |
| 10 | 6 | 40.48822664 | 47.5 | 1.96 |
